# In vitro evaluation of digestive and endolysosomal enzymes to cleave CML-modified Ara h 1 peptides

**DOI:** 10.1002/fsn3.215

**Published:** 2015-03-12

**Authors:** Christopher P Mattison, Jens Dinter, Matthew J Berberich, Si-Yin Chung, Shawndrika S Reed, Sylvie Le Gall, Casey C Grimm

**Affiliations:** 1 United States Department of Agriculture – Agricultural Research Service – Southern Regional Research Center1100 Robert E. Lee Blvd., New Orleans, Louisiana, 70124; 2 Ragon Institute of MGH, MIT and Harvard400 Technology Square, Cambridge, Massachusetts, 02139; 3 Harvard Medical SchoolBoston, Massachusetts

**Keywords:** Ara h 1, carboxymethyl lysine, Maillard reaction, mass spectrometry, peptide, proteolysis

## Abstract

Ara h 1 is a major peanut allergen. Processing-induced modifications may modulate the allergenic potency of Ara h 1. Carboxymethyl lysine (CML) modifications are a commonly described nonenzymatic modification on food proteins. In the current study, we tested the ability of digestive and endolysosomal proteases to cleave CML-modified and unmodified Ara h 1 peptides. Mass spectrometric analyses of the digested peptides demonstrate that carboxymethylation of lysine residues renders these peptides refractory to trypsin digestion. We did not detect observable differences in the simulated gastric fluid or endolysosomal digestion between the parental and CML-modified peptides. One of the tested peptides contains a lysine residue previously shown to be CML modified laying in a previously mapped linear IgE epitope, but we did not observe a difference in IgE binding between the modified and parental peptides. Our findings suggest a molecular mechanism for the increased resistance of peanut allergens modified by thermal processing, such as Ara h 1, to digestion in intestinal fluid after heating and could help explain how food processing-induced modifications may lead to more potent food allergens by acting to protect intact IgE epitopes from digestion by proteases targeting lysine residues.

## Introduction

Food allergies present a significant medical risk and have a negative impact on the quality of life for those affected by this condition. Annual costs associated with physician and ER visits associated with food allergies are estimated to be at around 25 billion dollars (Gupta et al. 2013), and recent studies indicate that the number of people with food allergies is increasing (Branum and Lukacs 2009; Sicherer et al. 2010; Gupta et al. 2011). Food allergen research has focused on the characterization of allergens in an effort to more clearly define the specific characteristics differentiating allergenic from nonallergenic food components, and to determine how food processing methods can enhance or reduce the ability of food allergens to cause food allergies.

Peanuts are among a group of eight foods that account for most allergic reactions (Sampson 2004). Peanut allergies affect an estimated 1% of the adult population (Sicherer et al. 2010). Peanut allergies are rarely outgrown, and they commonly cause severe reactions. While recent therapeutic advances are encouraging, there is discord among researchers regarding their therapeutic implementation (Wasserman et al. 2014; Wood and Sampson 2014), and the only widely accepted course of action for peanut allergies currently is avoidance. Although 10 peanut allergens have been identified to date, three of them (i.e., Ara h 1, 2, and 3) are most commonly bound by serum IgE (Koppelman et al. 2004). These proteins have had their linear IgE epitopes mapped (Burks et al. 1997; Stanley et al. 1997; Bush et al. 1998; Rabjohn et al. 1999), and they have been characterized structurally (Maleki et al. 2000b; Jin et al. 2009; Cabanos et al. 2011; Mueller et al. 2011).

Ara h 1 is a 64 kDa seed storage glycoprotein (Kolarich and Altmann 2000) representing approximately 12–16% of peanut protein content (Koppelman et al. 2001), has been identified as part of a 14S acylating enzyme complex (Parthibane et al. 2011), and binds proanthocyanidins (van Boxtel et al. 2007). Ara h 1 is a member of the functionally diverse cupin superfamily of proteins, it has a conserved beta-barrel fold, and is capable of forming trimers and higher molecular weight complexes (Maleki et al. 2000b; van Boxtel et al. 2006). Further, there is evidence that Ara h 1 can associate with Ara h 3 in a large complex (Boldt et al. 2005). Two reports indicate that the core residues of Ara h 1 form a conserved cupin fold that is sufficient for multimer formation in solution (Cabanos et al. 2011; Chruszcz et al. 2011).

The response to distinct peanut allergens by allergic individuals can vary greatly and is the result of several complex factors, including genetics, geography, sensitivity threshold, and food processing methods (Beyer et al. 2001; Wensing et al. 2002; Hong et al. 2009; Vereda et al. 2011). Several reports have provided differing views on the immunological consequences of heating peanuts, peanut extracts, and purified peanut allergens, depending upon the methods employed (Koppelman et al. 1999; Maleki et al. 2000a; Chung and Champagne 2001; Gruber et al. 2005; Mondoulet et al. 2005; Blanc et al. 2011; Mattison et al. 2011, 2012; Vissers et al. 2011a,b). During food preparation, allergenic proteins can be denatured, form aggregates due to cross-linking, precipitate, and undergo chemical modifications which can enhance their allergenic potential (Mills et al. 2009).

Advanced glycation end products (AGEs) are common nonenzymatic chemical modifications that occur in food proteins when they are stored or cooked. AGEs are a product of the Maillard reaction that can occur between sugars and proteins (Henle 2005), and AGEs contribute to aging-related degeneration and disease (Semba et al. 2010). AGEs, including carboxymethyl-lysine (CML), malondialdehyde (MDA), argpyrimidine (AP), and hydroxynonenal (HNE), have been characterized in peanut and almond extracts (Chung and Champagne 1999, 2001; Zhang et al. 2011; Mattison et al. 2012). Peanut allergen modifications have been identified by several groups and include CML modification on lysine residues (K287 and K547) of the peanut allergen Ara h 1 (Chassaigne et al. 2007; Mattison et al. 2011, 2012; Mueller et al. 2013).

Allergen processing in the digestive tract and in antigen processing cells plays an important role in the allergenicity of food proteins. Earlier reports suggested a correlation between stability to digestion and allergenicity (Astwood et al. 1996), but further research has suggested the correlation is not rigid (Fu et al. 2002; Thomas et al. 2004; Lucas et al. 2008). Some food allergens, such as 2S albumins from nuts, can cross the intestinal barrier intact and this can affect downstream antigen processing (Moreno et al. 2006; Price et al. 2013). Exogenous proteins can be transported across the epithelial barrier through intestinal M cells and can be internalized through phagocytosis, macropinocytosis, or endocytosis into endolysosomal vesicles (Price et al. 2013). Antigen processing involves several types of proteases and peptidases that can be localized in different subcellular compartments (Rucevic et al. 2014). In the cytosol, most proteolysis is mediated by the proteasome before antigens translocate into the endoplasmic reticulum for loading onto MHC-I molecules and presentation to CD8 T cells (Blum et al. 2013). Immunoproteasomes are specialized proteasomes containing stress inducible catalytic subunits that bias peptide generation to those containing hydrophobic carboxy-terminal residues (Basler et al. 2013; Kish-Trier and Hill 2013). Exogenous proteins that are internalized by professional antigen-presenting cells, such as dendritic cells, are degraded mainly by cathepsins in endolysosomes before loading onto MHC-II for presentation to CD4 T cells or escape into the cytosol for further processing by cytosolic proteases and subsequent cross-presentation onto MHC-I. A recent article has demonstrated the utility of crude lysates from peripheral blood mononuclear cells (PBMCs) in characterizing endolysosomal protease activity during antigen processing (Vaithilingam et al. 2013).

While the immunologic effect of food processing on specific food allergens may vary among individuals, it is clear that the allergenic potential of food allergens can be altered during thermal processing. For example, the consumption of cookies made with pecans caused anaphylaxis and subsequent analyses identified IgE binding that was specific for heated pecan proteins (Malanin et al. 1995). Further, there is evidence that thermal processing may modify the interaction between food allergens and cells of the intestinal lining (Starkl et al. 2011). Recently, modification of ovalbumin with pyrraline, an AGE, was shown to lead to increased immunogenicity as measured by enhanced dendritic cell uptake and IgE production (Heilmann et al. 2014a). One molecular explanation for the increased stability of food allergens is the formation of nonnative, or maintenance of inherent, disulfide bonds during heating (Mills et al. 2009). However, questions remain regarding the specific role of AGE modifications on the modulation of immunological properties of allergenic food components. In the current study, we evaluate the role CML modifications may play in gastric, intestinal, and endolysosomal protein digestion using synthetic Ara h 1 peptides.

## Materials and Methods

### Materials

Pepsin from porcine gastric mucosa and trypsin from bovine pancreas were purchased from Sigma. Sequencing grade-modified trypsin was purchased from Promega. Water was obtained using a Synergy Ultrapure water system (Millipore). Modified and unmodified Ara h 1 peptides were synthesized (at least 95% pure) and purchased from New England Peptide. Serum samples from five peanut allergic patients were purchased from PlasmaLab International (Everett, WA).

### Study participants

As described (Vaithilingam et al. 2013), human buffy coats from anonymous healthy donors were purchased at Massachusetts General Hospital and Partners Human Research Committee approved the use of the buffy coats under protocol no. 2005P001218.

### Peptide digestion

Simulated gastric fluid (SGF) containing pepsin and simulated intestinal fluid (SIF) containing trypsin were used as described previously (Mattison et al. 2014b). Synthetic peptides were resuspended in water just prior to use at a concentration of 1 mg/mL. Peptides (200 μg) were digested with SGF or SIF in 1 mL reaction volume for 1, 2, 4, or 16 h at 37°C. Samples (100 μL) were removed at the indicated time points, 5% formic acid was added, and samples were frozen at −80°C prior to analysis by mass spectrometry.

Cytosolic and endolysosomal digestion of peptides was performed and analyzed as described in Vaithilingam et al. (2013). The degradation assays in cytosol and endolysosomes were developed using postnuclear cell extracts at pH7.4 or pH4. It has been demonstrated that only cytosolic or endolyososomal peptidases are active at pH7.4 or pH4, respectively. They recapitulate the degradation in purified cytosol or endolysosomes but require fewer cells for preparation (Vaithilingam et al. 2013). About 4 nmol of Ara h 1 peptides were digested with 30 *μ*g of cell extracts, normalized to actin levels, at 37°C in 50 *μ*L of degradation buffer at pH7.4 or pH4 (50 mmol/L Tris-HCl, 137 mmol/L potassium acetate, 1 mmol/L MgCl_2_, and 1 mmol/L ATP, pH7.4, pH5.5, or pH4.0) (Vaithilingam et al. 2013). At various time points, the reaction was stopped with 2.5 *μ*L of 100% formic acid (FA) and peptide fragments were purified by 5% trichloroacetic acid precipitation.

### Patient Sera and IgE binding analysis

Competitive ELISA was used to evaluate IgE binding to Ara h 1 peptides with serum samples from peanut allergic individuals as described previously (Chung and Champagne 2007). Briefly, serum samples were diluted 1:20 and incubated with 2–2000 μg/mL peptide prior to incubation in a microtiter plate coated with peanut extract. Immune-complexes were probed with mouse anti-human IgE conjugated to horseradish peroxidase, and IgE binding was quantified with *o*-phenylenediamine dihydrochloride and scanning at 492 nm.

### Reverse phase high performance liquid chromatography mass spectrometry (RP-HPLC-MS) protein mass spectrometry

Analysis of trypsin/pepsin digestions: trypsin or pepsin digested Ara h 1 peptides were analyzed via liquid chromatography with tandem mass spectrometry (LC/MS/MS), using an Agilent 1200 LC system, an Agilent Chip Cube interface, and an Agilent 6520 Q-TOF tandem mass spectrometer (Agilent Technologies, Santa Clara, CA) as described in Mattison et al. (Mattison et al. 2014a). One microliter sample aliquots were transferred to an enrichment column via capillary pump operating at a flow of 4 *μ*L/min. The Nano pump was operated at a flow rate of 600 nL/min. An initial gradient (Solvent A-100% H_2_O, 0.1% Formic Acid; Solvent B- 90% ACN, 10% H_2_O and 0.1% Formic Acid) of 97% A was changed to 10% Solvent A at 2 min, 70% at 15 min, 100% at 20 min, and back to 97% at 25 min. A postrun time of 5 min was employed for column equilibration. An initial MS scan was performed from m/z 300 to 1600 and up to six multiply charged ions were selected for MS/MS analysis. The raw MS/MS data files were extracted, sequenced, and searched against a custom database containing the Ara h 1 sequence using Spectrum Mill software (Agilent Technologies, Santa Clara, CA). For relative quantification, samples were run in MS-only mode and chromatographic peak areas were integrated into selected ions representative of the peptide cleavage products, resulting from the protease digest.

Analysis of endolysosomal degradations: Equal amounts of cytosolic or endolyosomal peptide degradation samples were injected into a Nano-HPLC (Eksigent, Framingham MA) and online nanosprayed into an Orbitrap mass spectrometer (LTQ Orbitrap Discovery, Thermo, Waltham MA) with a flow rate of 400 nL/min. A Nano cHiPLC trap column (200 *μ*m × 0.5 mm ChromXP c18-CL 5 *μ*m 120Å; Eksigent, Framingham MA) was used to remove salts in the sample buffer. Peptides were separated in a Nano cHiPLC column (75 *μ*m × 15 cm ChromXP c18-CL 5 *μ*m 300Å; Eksigent) over a gradient of 2% to 40% buffer B (buffer A: 0.1% FA in water; buffer B: 0.1% FA in acetonitrile) and mass spectra were recorded in the range of 370 to 2000 Daltons. In tandem MS/MS mode, the eight most intense ions were selected with a window of 1 Da and fragmented. The collision gas was helium, and the collision voltage was 35V. Masses in the mass spectra were searched against source peptide databases with Proteome Discoverer (Thermo Scientific, Waltham MA). The integrated area under a peptide peak is proportional to its abundance (Zhang et al. 2012; Kourjian et al. 2014). We compared the degradation patterns and relative amount of peptides generated at each time point during the degradation in endolysosomal and cytosolic cell extracts. Each peptide is identified by LC-MS/MS by its sequence, charges and area under peak. The sum of all areas of all degradation peptides at a given time point corresponds to the total intensity. Peptides were grouped according to their lengths, and the contribution of each category of peptides to the total intensity of degradation products was calculated for each time point. Each sample was run on the mass spectrometer and analyzed at least twice.

### Ara h 1 modeling and alignments

Nonenzymatically induced modification sites were visualized onto the crystal structure of a shortened recombinant form of the Ara h 1 (rsAra h 1) monomer (Chruszcz et al. 2011), using Chimera software (Pettersen et al. 2004). Protein sequence alignments were performed using the COBALT multiple alignment tool at NCBI (Papadopoulos and Agarwala 2007).

## Results

### SGF and SIF digestion of Ara h 1 peptides-containing CML modification

Two peptides corresponding to sites of CML-modification previously described within Ara h 1(Chassaigne et al. 2007; Mattison et al. 2011, 2012; Mueller et al. 2013) were synthesized. One peptide included residues 285–307 (^285^VAKISMPVNTPGQFEDFFPASSR^307^, VAK) and the other residues 541–556 (^541^IFLAGDKDNVIDQIEK^556^, IFLAG) of Ara h 1. Both peptides were also synthesized with a CML modification, VAK-CML at lysine 287 (K287) and IFLAG-CML at lysine 547 (K547), with approximately 95% purity. The IFLAG peptide-containing residues 541–556, harbors the previously mapped linear IgE epitopes #19 and part of #20 (Shin et al. 1998), and mutation of the K547 lysine residue has been shown to result in the loss of IgE binding in epitope #19 (Shin et al. 1998). Competitive ELISA with serum from peanut allergic patients to assess IgE binding to each of these peptides did not reveal observable differences in binding due to the presence of the CML modification (data not shown).

**Figure 1:**
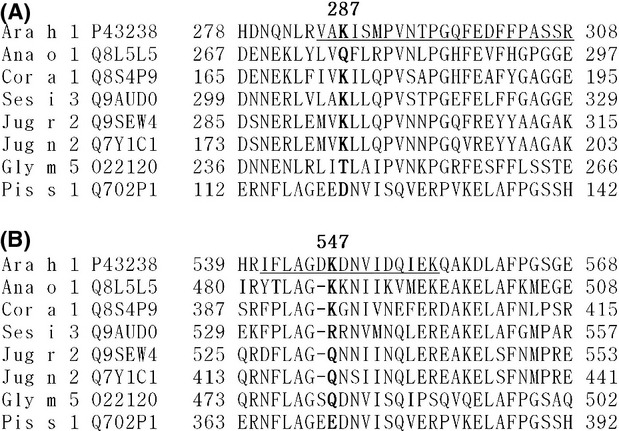
Conservation of CML-modified Ara h 1 residues among vicilins. The sequence of Ara h 1 was aligned with other 7S vicilins (cashew Ana o 1, hazelnut Cor a 1, sesame Ses i 3, English walnut Jug r 1, black walnut Jug n 1, soybean Gly m 5, and pea Pis s 1) using COBALT and the segment of the resulting alignment corresponding to residues 278–308 (A) or 539–568 (B) of Ara h 1 from each of the proteins was extracted to highlight this region of the proteins. Residues K287 and K547 are bolded to highlight conservation and peptides synthesized for this work are underlined.

Both the K287 and K547 residues are relatively well conserved among seed storage vicilins (Fig.1). The carboxyl terminus of several vicilins is conserved (Barre et al. 2008), and our alignment shows the K547 residue is conserved among vicilins from cashew Ana o 1 and hazelnut Cor a 1, and there is charge conservation at the corresponding arginine residue in the sesame Ses i 3 protein (Fig.1). The more amino-terminal K287 residue is conserved among hazelnut, sesame, and walnut vicilins.

To characterize the effect these modifications could have during digestion, we evaluated the ability of simulated gastric fluid to digest the unmodified and CML-modified Ara h 1 peptides in vitro. We found no difference between parental or CML-modified peptides following SGF (pepsin) digestion. Mass spectrometric analysis of the peptides resulting from SGF digest revealed no observable difference in the cleavage of the peptides (Table1). The peptide cleavage product containing the CML modification was detected at equivalent intensities, indicating the CML modification did not affect the ability of pepsin to digest the peptide. After 16 h both modified and unmodified peptides were digested to near completion, with only a small % remaining undigested.

**Table 1:** Major peptide cleavage products following 16 h *in vitro* SGF digestion

Peptide sequence	% Detected^1^	
	Unmodified	CML modified
IFLAGD**K**DNVIDQIEK	3	2
IFLAGD**K**DNVIDQ	57	52
AGD**K**DNVIDQ	10	14
IFLAGD**K**D	30	32
VA**K**ISMPVNTPGQFEDFFPASSR	0	1
VA**K**ISMPVNTPGQF	74	80
VA**K**ISM	26	19

1 Semiquantitative analysis of cleavage products was accomplished by comparing relative peak areas of identical ions between samples.

**Figure 2:**
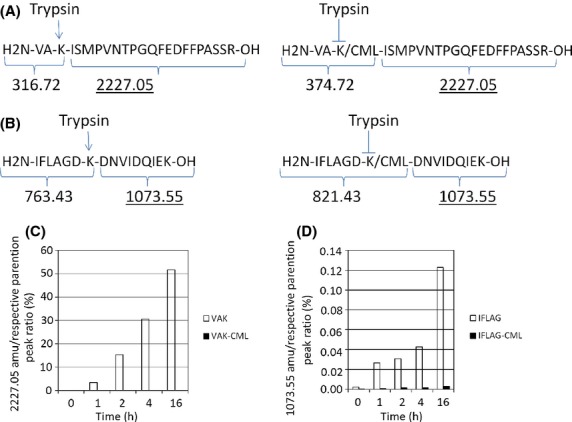
Carboxymethyl lysine-modification of K287 or K547 within Ara h 1 peptides prevents cleavage by trypsin *in vitro*. Diagram of synthesized peptide sequences (^285^VAKISMPVNTPGQFEDFFPASSR^307^ (A) and ^541^IFLAGDKDNVIDQIEK^556^ (B)) and expected masses of resulting cleavage products following digestion by trypsin in SIF. Plots of the ratio of observed 2227.05 amu (C) and 1073.55 amu (D) fragment ions over the total accumulated intensities of +2 and +3 charge species of fragment and parent ions following peak integration.

We evaluated the digestion of the modified and unmodified peptides using SIF containing only trypsin to mimic intestinal digestion conditions. The trypsin in SIF cleaves at the C-terminal side of lysines and arginines, and the CML modifications in our modified peptides would be expected to prevent cleavage by trypsin. Trypsin digestion of the unmodified VAK and IFLAG peptides results in cleavage products of 316.72 and 2227.05 and 763.43 and 1073.55, respectively (Fig.2A and B). If cleavage occurs in the CML-modified VAK or IFLAG peptides, cleavage products of 374.72 and 2227.05 and 821.43 and 1073.55 would be expected (Fig.2A and B). As a measure of trypsin activity, we followed the formation of the 2227.05 amu ion for the VAK peptide and 1073.55 amu ion for the IFLAG peptide. To quantify the results, we integrated the relative peak areas of the 2227.05 and 1073.55 amu ions and undigested parental ions, and plotted the generation of the peptide cleavage products as a percentage of the sum of the parental and fragment ion values over time (Fig.2C and D). As shown in Figure2C, the unmodified VAK peptide was a good SIF substrate and about 50% of the peptide was cleaved at the K287 site resulting in formation of the 2227.05 amu ion (Fig.2C). In contrast, less than 5% of the CML-modified VAK peptide was cleaved. The unmodified IFLAG peptide was a poor substrate and we detected very limited SIF proteolysis. However, we observed similar results and found that while the presence of the 1073.55 amu cleavage ion increased over time in the unmodified peptide sample, the CML modification prevented trypsin cleavage of the peptide (Fig.2D). The CML-modified peptides were approximately 95% pure and the small increases in cleavage observed in the CML-modified peptides can be attributed to the presence of 5% of the unmodified form.

### Cytosolic and endolysosomal digestion of Ara h 1 peptides-containing CML modification in human PBMC cell extracts

**Figure 3:**
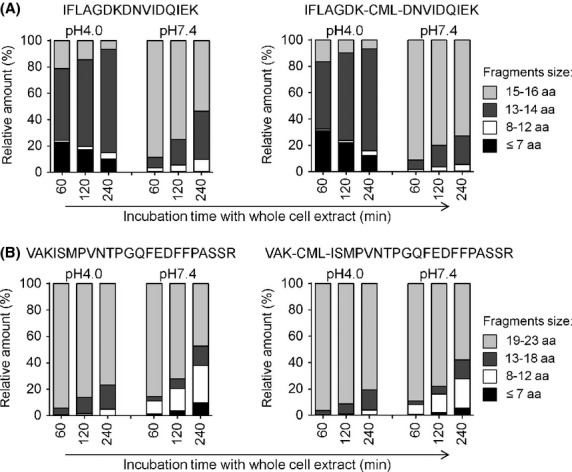
Cytosolic and endolysosomal degradation of Ara h 1 peptides in whole cell extracts from human PBMCs. Cleavage of the peptides is represented as a relative percentage of the total material detected by mass spectrometry. IFLAG and IFLAG-CML peptides (IFLAGDKDNVIDQIEK, aa 541–556 in Ara h 1, A) were degraded in whole cell extracts from human PBMCs for 60, 120, and 240 min. Degradation products identified by mass spectrometry were grouped according to their length of cleavage products: 15–16 aa (light gray), 13–14 aa (gray), 8–12 aa (white), and cleavage products equal or shorter than 7 aa (black). VAK and VAK-CML peptides (VAKISMPVNTPGQFEDFFPASSR, aa 285–307 in Ara h 1, B) were degraded as described in A. Degradation products identified by mass spectrometry were grouped according to their length of cleavage products: 19–23 aa (light gray), 13–18 aa (gray), 8–12 (white) and cleavage products equal or shorter than 7 aa (black). The peak area of each identified peptide was calculated with Proteome Discoverer, and the contribution of each category of peptides to the total intensity of all degradation products is shown at each time point. Data are representative of 2 independent degradation experiments.

To determine what effect CML modification of the Ara h 1 peptides has on degradation by cytosolic and endolysosomal peptidases in human primary cells, peptides were subjected to incubation in crude PBMC lysates, and degradation products characterized by LC-MS/MS. All 4 peptides were only very slowly degraded over 4 h regardless of CML modification, indicating a very high stability against degradation by intracellular peptidases (Fig.3A and B). The IFLAG peptides were more quickly degraded by endolysosomal peptidases (pH 4.0) compared with cytosolic peptidases (pH 7.4), whereas the opposite effect was observed for the two VAK peptides. Further analyses of the cleavage sites within all 4 peptides by compartment-specific peptidases demonstrated a preferential trimming from the amino terminus, while the carboxy terminus was relatively stable. In contrast to the analysis of HIV-derived peptides with similar lengths using this assay (Vaithilingam et al. 2013; Dinter et al. 2014), the peanut Ara h 1 peptides we evaluated were extremely stable in the cytosol and in endolysosomal compartments of PBMC.

### Visualization and conservation of modified residues

**Figure 4:**
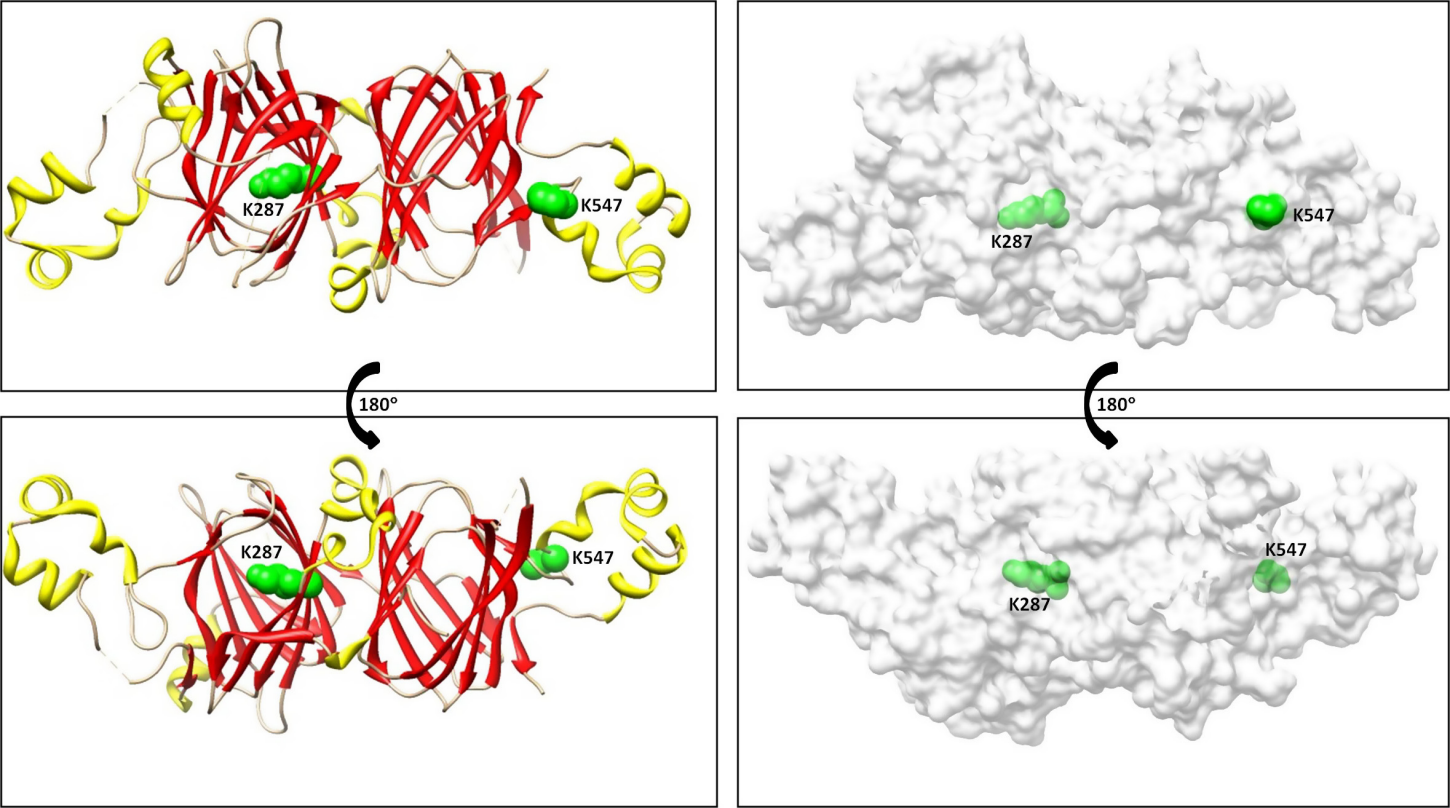
Ribbon and space-filling models featuring the CML modification sites. The sites of CML modification (K287 and K547) are highlighted in green on a recombinant short form of Ara h 1 from (Chruszcz et al. 2011). The bottom panel is rotated through 180°.

We highlighted the modified residues on the recently determined structure of a shortened recombinant Ara h 1 molecule (Chruszcz et al. 2011) (Fig.4). The K547 residue lies within a surface exposed C-terminal α-helix, while K287 lies within a conserved β-barrel core domain that is completely buried within the protein structure. The K287 residue is amino-terminal to the previously mapped epitope 10 (Shin et al. 1998) and the buried position of this lysine suggests that modification at this residue may occur after protein unfolding or degradation has increased solvent accessibility. In contrast to the buried positioning of the K287 residue, the K547 residue is surface exposed in both the monomeric and trimeric forms of the protein (Cabanos et al. 2011; Chruszcz et al. 2011). The carboxy-terminal region of the protein containing the CML-modified K547 peptides also contains linear IgE epitopes 19, 20 and 21 (Shin et al. 1998) and has been highlighted as a surface exposed region that is conserved among vicilin allergens (Barre et al. 2008). In particular, the K547 residue within epitope 19 protrudes from the surface of the protein (Barre et al. 2008), and may be a preferred site for modification due to its physiochemical context within the protein. Due to sequence and structural conservation among vicilin proteins, these conserved lysine residues are potential modification sites within these and possibly other vicilins in foods.

## Discussion and Conclusions

A hallmark of food allergens is stability during digestion (Astwood et al. 1996), but the correlation is not absolute (Fu et al. 2002; Thomas et al. 2004; Lucas et al. 2008). Stability to digestive enzymes may allow intact proteins to reach the intestinal lining and be absorbed intact. Primary sequence, structural cues, as well as other factors such as protein modification can affect the ability of food allergens to be digested. The structure of Ara h 1 has been suggested to protect it from degradation (Maleki et al. 2000b), and there is a correlation between thermally processed Ara h 1-containing modified lysine and arginine residues and the increased stability it displays (Maleki et al. 2000a; Chung and Champagne 2001). We and others have noticed that several of the processing-induced modifications observed in food allergens were from peptides containing a missed lysine or arginine cleavage event at the site of the modification. This distinction has been used to aid in the identification of CML and CMR modifications in RNase (Brock et al. 2003, 2007; Cotham et al. 2004).

Our results provide a mechanism by which the modification of lysine and possibly arginine residues, by the Maillard reaction during peanut processing, can act to stabilize antigenic peptides against digestion by intestinal trypsin and thereby preserve IgE epitopes. Our findings are generally applicable to other food allergens, and provide a clear molecular mechanism explaining how similar modifications induced by processing on lysine or arginine residues may act to preserve protein structure and IgE-binding epitopes within food allergens by preventing their digestion depending upon the specific peptide. Nonenzymatic processing events on food allergens likely affect their gastric and intestinal digestion, but may also play a role in their antigenic processing. Even if only a small amount of modified allergen was present, its stability to intestinal digestion could lead to more of that form of the protein-surviving digestion and priming the immune system. AGEs such as pyrraline have been shown to enhance the T-cell immunogenicity of ovalbumin by promoting uptake in dendritic cells (Heilmann et al. 2004b).

Other mass spectrometry studies have characterized the processing-induced changes in the peptide profile of proteins from peanut extracts and foods and proposed the utility of specific peptides as markers for the presence of peanuts (Shefcheck and Musser 2004; Shefcheck et al. 2006; Chassaigne et al. 2007). Allergen peptides can be used as diagnostic markers and similar studies have identified the partially digested 541-IFLAGDKDNVIDQIEK-556 Ara h 1 peptide as a marker of interest. This peptide was designated a diagnostic marker for the presence of peanut in foods (Shefcheck and Musser 2004) and a segment of the peptide-containing residues 541–553 was observed in the modified form only in strongly roasted peanut extracts (Chassaigne et al. 2007). However, we have observed the CML-modified form of the 541-IFLAGDKDNVIDQIEK-556 peptide in raw and roasted peanut extracts (Mattison et al. 2011, 2012).

We demonstrate that CML modification within a previously mapped Ara h 1 linear IgE epitope renders the site resistant to digestion by SIF leaving the epitope intact. The accumulation of processing-induced lysine modifications within the carboxy-terminus and the resulting resistance to digestion could increase the stability of this part of the protein as it passes through the digestive system and preferentially preserve this segment of the protein for antigenic processing in endolysosomes. The presence of these modifications and their increased stability may help explain the concentration of several IgE epitopes in this segment of the protein. An intense area of research focuses on how protein modifications induced by heating effect IgE binding, and there are several conflicting reports on this topic (Koppelman et al. 1999; Maleki et al. 2000a; Chung and Champagne 2001; Gruber et al. 2005; Mondoulet et al. 2005; Blanc et al. 2011; Vissers et al. 2011a,b). While not a focus of this report, the question of whether the modifications we have analyzed can act to mask or contribute to recognition by IgE will require continued investigation. Continued work in this area will help define the role these and as yet unidentified processing-induced modifications play in allergen structural alterations, resistance to digestion, recognition by IgE, and absorption in the gut.
